# Dermohypodermite révélant une filariose

**DOI:** 10.11604/pamj.2015.20.344.6192

**Published:** 2015-04-10

**Authors:** Redouane Ouakrim, Mohamed Saleh Berrada

**Affiliations:** 1 Service de Chirurgie Orthopédique CHU de Rabat Maroc

**Keywords:** Filariose, dermohypodermite, calcifications, filariasis, dermohypodermitis, calcification

## Image en medicine

**Figure 1:**
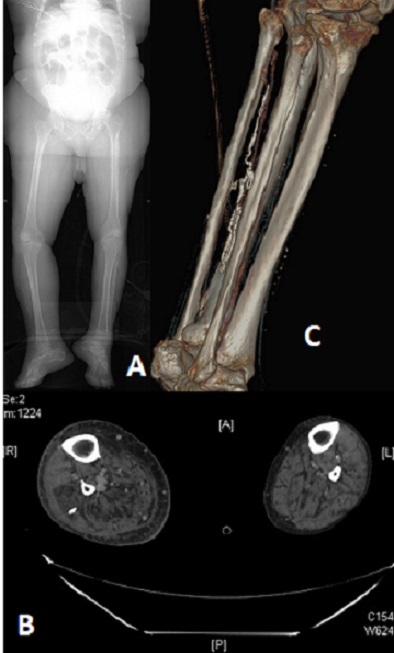
(A) pangonogramme montrant une augmentation du volume de la jambe droite avec calcifications; (B) TDM montrant une calcification externe droite; (C) reconstruction scanographique montrant la filariose en 3D

Il s'agit d'un patient de peau noir admis au service des urgences, pour une dermohypodermite du membre inferieur droit. Le traitement initial à base d'amoxilline protéger n'a pas donné de résultat efficace. L'aggravation clinique nous a poussé faire une TDM du membre, qui nous a montré un aspect de calcification filiforme. Des incisions de décharge, associée à une antibiothérapie de plus large spectre ont permit la résolution du problème. La filariose de Médine, est une maladie parasitaire causée par un nématode. ce ver appelé Dracunculus medinensis. Le nom de Dracunculus vient du latin «petit dragon». Il est présent dans des crustacés, les cyclopes, vivant dans l'eau stagnante. La femelle est le plus gros parasite qui puisse se loger dans les tissus humains, elle peut mesurer jusqu’à un mètre de long. Lorsqu'elle est fécondée, son corps n'est plus qu'un long sac s'ouvrant seulement par la bouche et occupé presque entièrement par l'utérus bourré d'embryons. Le métronidazole ou le thiabendazole (chez les adultes) est habituellement utilisé en complément de la méthode du bâton et facilite légèrement le processus d'extraction. Cependant, une étude a constaté que le traitement anthelminthique était associé à une migration anormale des vers, ayant pour résultat l'infection dans d'autres zones que les membres inférieurs. Par conséquent, de tels médicaments devraient être utilisés avec prudence. Le ver peut également être excisé chirurgicalement quand les équipements nécessaires sont disponibles. Chez notre patient la filariose était chronique, et elle a joué un support d'entretien de l'infection. Figure 1

